# Supplementary material for: Exceptional parallelisms characterize the evolutionary transition to live birth in phrynosomatid lizards
Source: Nat Commun. 2022 May 24;13:2881. doi: 10.1038/s41467-022-30535-w (PMC9130271; doi:10.1038/s41467-022-30535-w)
Supplement: Supplementary file 1 — Supplementary Information [file 41467_2022_30535_MOESM1_ESM.pdf]

Supplementary information

Exceptional parallelisms characterize the evolutionary transition to live birth in phrynosomatid lizards

Saúl F. Domínguez-Guerrero[1,2,3*], Fausto R. Méndez-de la Cruz[2], Norma L. Manríquez-Morán[4], Mark E. Olson[2], Patricia Galina-Tessaro[5], Diego M. Arenas-Moreno[2,3], Adán Bautista-del Moral[2,3], Adriana Benítez-Villaseñor[2,3], Héctor Gadsden[6], Rafael A. Lara-Reséndiz[5,7], Carlos A. Maciel-Mata[4], Francisco J. Muñoz-Nolasco[2,3], Rufino Santos-Bibiano[2,3], Jorge H. Valdez-Villavicencio[8], Guillermo A. Woolrich-Piña[9] and Martha M. Muñoz[1].

This document includes:

***Supplementary Figures*……………………………………………………………..…2**

Supplementary Figure 1……………………………………………………...2

Supplementary Figure 2……………………………………………………...4

Supplementary Figure 3……………………………………………………...5

Supplementary Figure 4……………………………………………………...6

***Supplementary Tables*..7**

Supplementary Table 1……………..…………………………………………7

Supplementary Table 2……………..……………………………...…………9

Supplementary Table 3…………..……………………………………….……11

Supplementary Table 4……………………………………………….……..13

Supplementary Table 5……………………………………………….……..16

Supplementary Table 6……………………………………………….……..17

Supplementary Table 7……………………………………………………...18

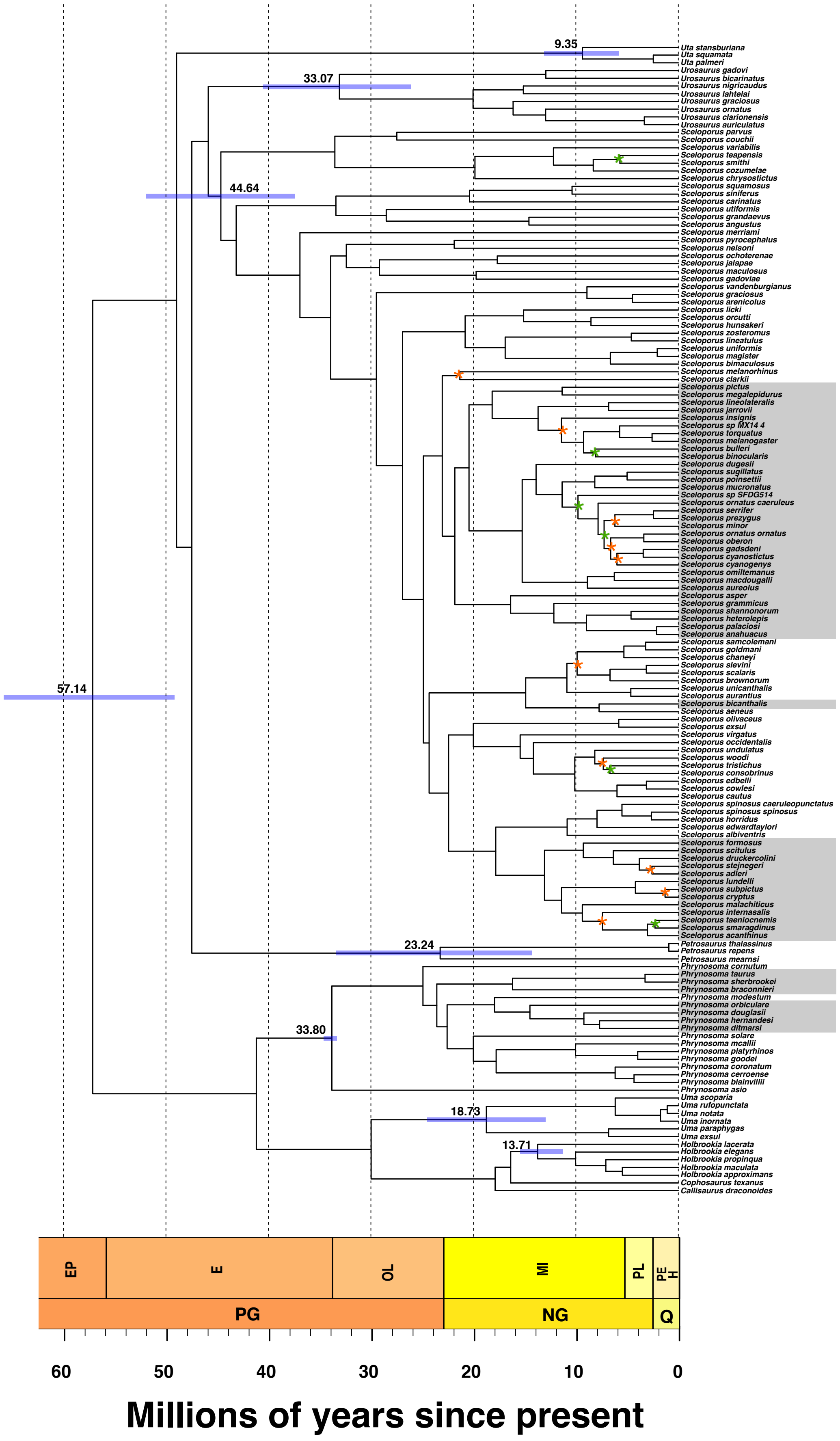
9.35
33.07
44.64
57.14
23.24
33.80
18.73
13.71
Uta stansburiana
Uta squamata
Uta palmeri
Urosaurus gadovi
Urosaurus bicarinatus
Urosaurus nigricaudus
Urosaurus lahtelai
Urosaurus graciosus
Urosaurus ornatus
Urosaurus clarionensis
Urosaurus auriculatus
Sceloporus parvus
Sceloporus couchii
Sceloporus variabilis
Sceloporus teapensis
Sceloporus smithi
Sceloporus cozumelae
Sceloporus chrysostictus
Sceloporus squamosus
Sceloporus siniferus
Sceloporus carinatus
Sceloporus utiformis
Sceloporus grandaevus
Sceloporus angustus
Sceloporus merriami
Sceloporus pyrocephalus
Sceloporus nelsoni
Sceloporus ochoterenae
Sceloporus jalapae
Sceloporus maculosus
Sceloporus gadoviae
Sceloporus vandenburgianus
Sceloporus graciosus
Sceloporus arenicolus
Sceloporus licki
Sceloporus orcutti
Sceloporus hunsakeri
Sceloporus zosteromus
Sceloporus lineatulus
Sceloporus uniformis
Sceloporus magister
Sceloporus bimaculosus
Sceloporus melanorhinus
Sceloporus clarkii
Sceloporus pictus
Sceloporus megalepidurus
Sceloporus lineolateralis
Sceloporus jarrovii
Sceloporus insignis
Sceloporus sp MX14 4
Sceloporus torquatus
Sceloporus melanogaster
Sceloporus bulleri
Sceloporus binocularis
Sceloporus dugesii
Sceloporus sugillatus
Sceloporus poinsettii
Sceloporus mucronatus
Sceloporus sp SFDG514
Sceloporus ornatus caeruleus
Sceloporus serrifer
Sceloporus prezygus
Sceloporus minor
Sceloporus ornatus ornatus
Sceloporus oberon
Sceloporus gadsdeni
Sceloporus cyanostictus
Sceloporus cyanogenys
Sceloporus omiltemanus
Sceloporus macdougalli
Sceloporus aureolus
Sceloporus asper
Sceloporus grammicus
Sceloporus shannonorum
Sceloporus heterolepis
Sceloporus palaciosi
Sceloporus anahuacus
Sceloporus samcolemani
Sceloporus goldmani
Sceloporus chaneyi
Sceloporus slevini
Sceloporus scalaris
Sceloporus brownorum
Sceloporus unicanthalis
Sceloporus aurantius
Sceloporus bicanthalis
Sceloporus aeneus
Sceloporus olivaceus
Sceloporus exsul
Sceloporus virgatus
Sceloporus occidentalis
Sceloporus undulatus
Sceloporus woodi
Sceloporus tristichus
Sceloporus consobrinus
Sceloporus edbelli
Sceloporus cowlesi
Sceloporus cautus
Sceloporus spinosus caeruleopunctatus
Sceloporus spinosus spinosus
Sceloporus horridus
Sceloporus edwardtaylori
Sceloporus albiventris
Sceloporus formosus
Sceloporus scitulus
Sceloporus druckercolini
Sceloporus stejnegeri
Sceloporus adleri
Sceloporus lundelli
Sceloporus subpictus
Sceloporus cryptus
Sceloporus malachiticus
Sceloporus internasalis
Sceloporus taeniocnemis
Sceloporus smaragdinus
Sceloporus acanthinus
Petrosaurus thalassinus
Petrosaurus repens
Petrosaurus mearnsi
Phrynosoma cornutum
Phrynosoma taurus
Phrynosoma sherbrookei
Phrynosoma braconnieri
Phrynosoma modestum
Phrynosoma orbiculare
Phrynosoma douglasii
Phrynosoma hernandesi
Phrynosoma ditmarsi
Phrynosoma solare
Phrynosoma mcallii
Phrynosoma platyrhinos
Phrynosoma goodei
Phrynosoma coronatum
Phrynosoma cerroense
Phrynosoma blainvillii
Phrynosoma asio
Uma scoparia
Uma rufopunctata
Uma notata
Uma inornata
Uma paraphygas
Uma exsul
Holbrookia lacerata
Holbrookia elegans
Holbrookia propinqua
Holbrookia maculata
Holbrookia approximans
Cophosaurus texanus
Callisaurus draconoides
EP
E
OL
MI
PL
PE
H
PG
NG
Q
60
50
40
30
20
10
0
Millions of years since present

Supplementary Figure 1. Time-calibrated phylogeny for phrynosomatid lizards. Gray shading denotes viviparous species. Blue bars show the confidence interval for the estimated ages of crown groups and the mean ages in the phylogeny. Nodes without asterisks represent node values with posterior probability (PP) >0.8, orange asterisks represent node values with PP between 0.79- 0.5, and green asterisks represent node values with PP <0.5. Geologic periods are given by the following abbreviations: PG=Paleogene, NG=Neogene, and Q=Quaternary. Geologic epochs are given by the following abbreviations: EP=Paleocene, E=Eocene, OL=Oligocene, MI=Miocene, PL=Pliocene, PE=Pleistocene, and H=Holocene.

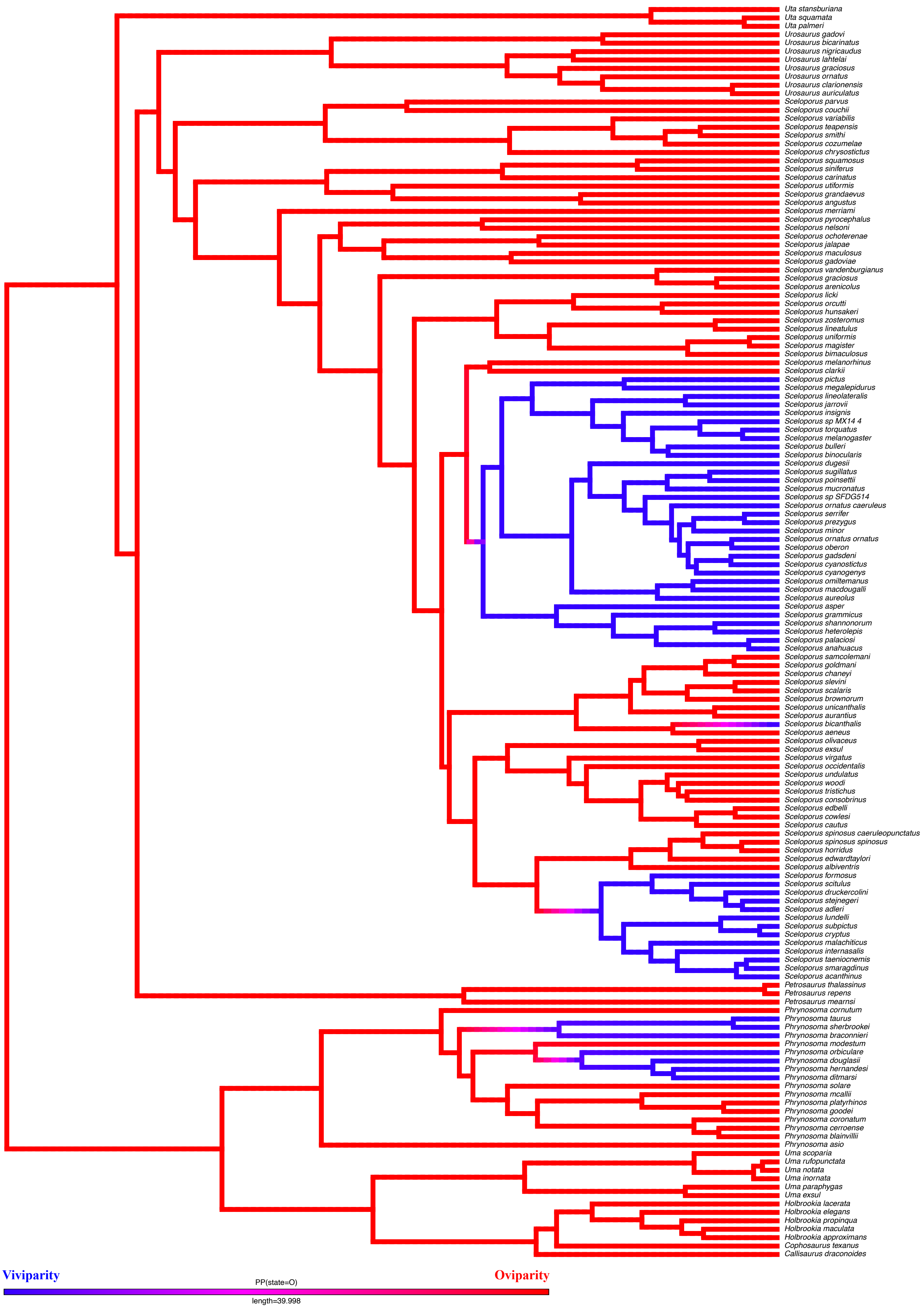

Supplementary Figure 2. Stochastic character mapping of parity mode across phrynosomatid lizards.

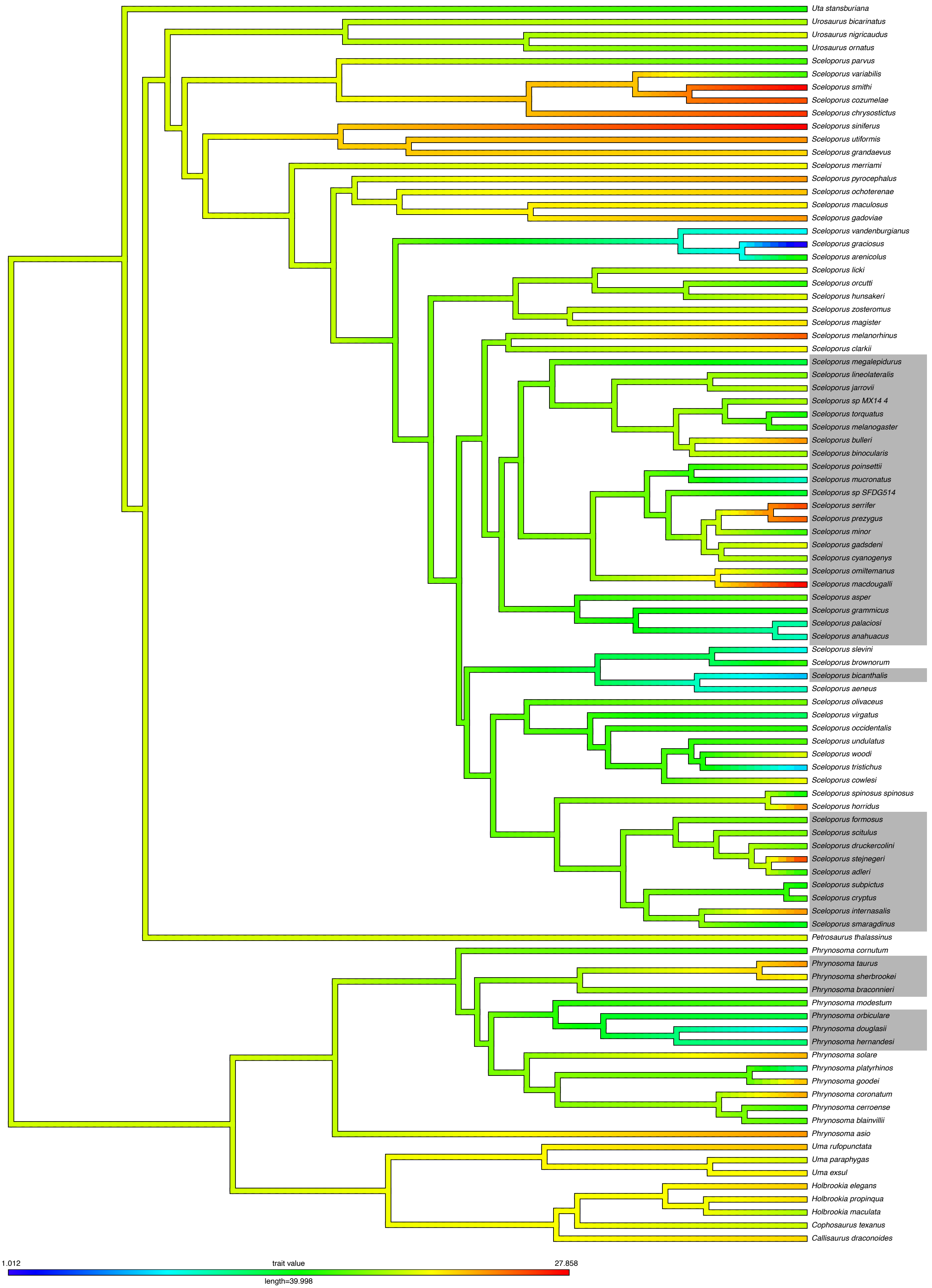

Supplementary Figure 3. Ancestral state reconstruction of mean annual temperature for phrynosomatid lizards. Gray shading corresponds to viviparous species.

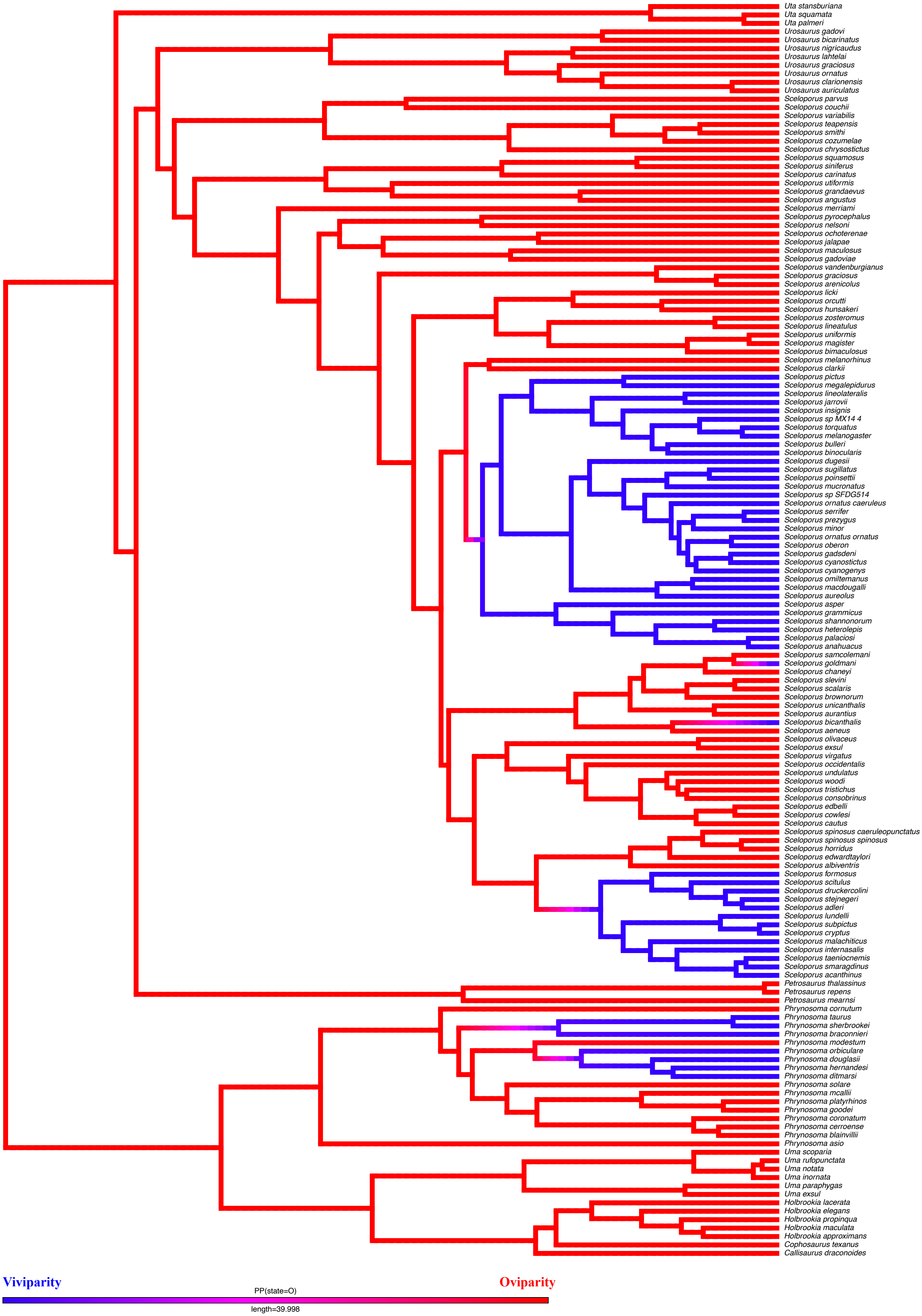

Supplementary Figure 4. Stochastic character mapping of parity mode across phrynosomatid lizards, considering to *Sceloporus goldmani* as a viviparous species.

Supplementary Table 1. Intersexual comparison of field body temperature (T_b), preferred body temperature (T_{pref}), critical thermal minimum (CT_{min}) and critical thermal maximum (CT_{max}) of phrynosomatid lizards. The physiological traits are given in degrees Celsius (±1s.e.). The last row shows two-sided *t*-tests comparing traits among sexes.

**Species**	**Mean T_b (*n*)**	**Mean T_{pref} (*n*)**	**Mean CT_{min} (*n*)**	**Mean CT_{max} (*n*)**
Callisaurus draconoides				
Females	39 ± 0.53 (16)	34.32 ± 1.03 (15)	12.45 ± 0.45 (2)	42.65 ± 1.05 (2)
Males	38.99 ± 0.73 (12)	35.11 ± 1.04 (12)	13.46 ± 0.68 (8)	43.22 ± 0.1 (5)
Petrosaurus thalassinus				
Females	36.39 ± 0.41 (11)	31.54 ± 0.67 (11)	17.07 ± 0.4 (10)	39.2 (1)
Males	34.28 ± 0.9 (9)	30.57 ± 1.16 (9)	-	39.66 ± 0.41 (8)
Phrynosoma cerroense				
Females	36.2 ± 0.75 (5)	35.4 ± 0.68 (8)	8 ± 0.3 (8)	42.1 ± 0.54 (4)
Males	37.2 ± 2.01 (3)	33.95 ± 0.29 (3)	7.6 ± 0.49 (2)	40.2 (1)
Phrynosoma orbiculare				
Females	31.89 ± 1.57 (10)	31.54 ± 0.7 (10)	4.82 ± 0.3 (9)	37.71 ± 0.28 (9)
Males	29.95 ± 1.58 (12)	31.21 ± 0.42 (12)	4.25 ± 0.33 (10)	37.94 ± 0.38 (10)
Phrynosoma sherbrookei				
Females	35.4 ± 2.6 (2)	33.74 ± 0.53 (2)	13.6 ± 0.2 (2)	-
Males	33.37 ± 0.76 (7)	37.23 ± 0.2 (7)	11.6 ± 0.53 (7)	43.13 ± 0.64 (7)
Sceloporus adleri				
Females	30.4 ± 0.58 (24)	32.85 ± 0.41 (20)	-	-
Males	31.45 ± 0.9 (17)	30.84 ± 2.82 (7)	9.56 ± 0.52 (7)	39.01 ± 0.74 (7)
Sceloporus brownorum				
Females	37.44 ± 0.6 (14)	33.96 ± 0.45 (13)	11.17 ± 0.57 (7)	42.4 ± 0.2 (2)
Males	33.45 ± 0.8 (6)	35.69 ± 0.24 (6)	11.75 ± 0.25 (2)	42.57 ± 0.03 (3)
Sceloporus bulleri				
Females	31.37 ± 1.35 (6)	32.46 ± 0.84 (9)	15.08 ± 1.08 (5)	37.5 ± 0.86 (4)
Males	32.62 ± 1.3 (6)	31.23 ± 0.79 (9)	15.48 ± 1.08 (4)	37.22 ± 0.6 (5)
Sceloporus grammicus				
Females	32.18 ± 0.42 (45)	32.89 ± 0.53 (49)	10.03 ± 0.35 (43)	39.97 ± 0.26 (34)
Males	31.73 ± 0.57 (35)	33.31 ± 0.32 (43)	10.54 ± 0.33 (34)	39.93 ± 0.28 (30)
Sceloporus hunsakeri				
Females	34.42 ± 0.7 (6)	34.5 ± 0.48 (3)	17.12 ± 0.84 (4)	38.95 ± 0.45 (2)
Males	33.75 ± 1.55 (2)	33.06 ± 1.62 (2)	-	39.95 ± 0.15 (2)
Sceloporus jarrovii				
Females	35.64 ± 0.66 (9)	32.04 ± 0.33 (7)	15.51 ± 0.42 (7)	39.8 ± 1.1 (3)
Males	35.37 ± 2.85 (3)	32.14 ± 0.44 (2)	11.5 ± 0.2 (2)	39.05 ± 1.45 (2)
Sceloporus licki				
Females	33.73 ± 0.95 (6)	32.21 ± 1.05 (6)	17.48 ± 0.45 (4)	38 ± 0.3 (2)
Males	33.1 ± 0.32 (3)	33.32 ± 0.77 (3)	15.9 (1)	40 ± 0.2 (2)
Sceloporus magister				
Females	38.3 ± 1.7 (2)	34.91 ± 0.65 (3)	10.55 ± 0.65 (2)	40.2 (1)

Males	33.3 (1)	34.84 ± 0.32 (2)	10.4 (1)	39.2 (1)
Sceloporus minor				
Females	27.47 ± 1.3 (10)	32.37 ± 0.33 (9)	10 ± 0.7 (5)	37.17 ± 0.62 (3)
Males	28.04 ± 1.71 (7)	32.74 ± 0.52 (7)	9.18 ± 0.81 (5)	37.17 ± 0.44 (7)
Sceloporus megalepidurus				
Females	28.26 ± 1.08 (7)	33.87 ± 0.48 (6)	9.03 ± 0.78 (3)	39.25 ± 0.35 (2)
Males	27.49 ± 1.07 (7)	33.49 ± 0.13 (7)	9.08 ± 0.41 (6)	39.37 ± 0.27 (7)
Sceloporus melanogaster				
Females	29.6 ± 0.79 (11)	32.8 ± 0.52 (11)	8.5 ± 0.28 (7)	38 ± 0.45 (6)
Males	33.9 ± 1 (2)	32.3 ± 0.53 (2)	8.7 ± 0.95 (2)	37.1 ± 1 (2)
Sceloporus mucronatus				
Females	30.44 ± 1.05 (10)	33.85 ± 0.38 (10)	5.28 ± 0.42 (6)	-
Males	32.61 ± 0.89 (7)	34.18 ± 0.58 (9)	7 ± 0.7 (3)	37.62 ± 0.49 (6)
Sceloporus occidentalis				
Females	34.35 ± 0.66 (14)	34.96 ± 1.03 (5)	8.5 (1)	37.1 (1)
Males	33.26 ± 0.51 (17)	32.28 ± 1.32 (10)	11.03 ± 0.46 (9)	38.72 ± 0.58 (5)
Sceloporus parvus				
Females	34 ± 0.4 (5)	31.47 ± 1.28 (5)	12.93 ± 0.84 (3)	39.9 (1)
Males	32.34 ± 0.63 (8)	33.32 ± 0.52 (9)	13.85 ± 0.71 (4)	40.42 ± 0.32 (5)
Sceloporus pyrocephalus				
Females	34.82 ± 0.47 (6)	33.57 ± 0.68 (5)	16.6 ± 0.4 (2)	-
Males	36.73 ± 0.65 (11)	33.9 ± 0.62 (10)	16.13 ± 0.41 (7)	41.21 ± 0.54 (7)
Sceloporus torquatus				
Females	33.31 ± 0.4 (54)	33.13 ± 0.31 (61)	9.1 ± 0.29 (46)	39.38 ± 0.27 (43)
Males	33.38 ± 0.36 (53)	33.24 ± 0.27 (55)	8.52 ± 0.27 (54)	39.08 ± 0.23 (45)
Sceloporus vandenburgianus				
Females	32.12 ± 0.55 (29)	33.83 ± 0.74 (10)	6.3 ± 0.7 (2)	-
Males	32.61 ± 0.31 (42)	34.75 ± 0.6 (14)	6.9 ± 0.36 (11)	38.83 ± 0.43 (10)
Sceloporus variabilis				
Females	26.15 ± 0.95 (8)	32.84 ± 0.37 (8)	10 ± 0.5 (3)	39.6 ± 0.49 (3)
Males	29.93 ± 0.72 (10)	34.92 ± 0.68 (6)	9.94 ± 0.3 (7)	41.1 ± 0.41 (7)
Sceloporus zoosteroumus				
Females	34.3 ± 1.18 (4)	33.11 ± 1.08 (3)	11.55 ± 0.55 (4)	-
Males	33.75 ± 0.91 (8)	34.3 ± 1.71 (6)	11.1 ± 1.1 (2)	40.68 ± 0.52 (5)
Uta stansburiana				
Females	32.66 ± 0.79 (13)	34.32 ± 0.41 (11)	9.73 ± 1.27 (3)	41.35 ± 0.55 (2)
Males	33.66 ± 1.24 (9)	35.3 ± 0.84 (6)	8.73 ± 0.76 (6)	40.03 ± 0.95 (3)
All (mean)				
Females	33.19 ± 0.66 (25)	33.3 ± 0.22 (25)	11.27 ± 0.76 (24)	39.49 ± 0.39 (19)
Males	33.05 ± 0.51 (25)	33.49 ± 0.32 (25)	10.53 ± 0.63 (23)	39.7 ± 0.34 (25)
***t*-tests**	***t*=0.172, *df*=48, *P*=0.86**	***t*=-0.482, *df*=48, *P*=0.63**	***t*=0.742, *df*=45, *P*=0.46**	***t*=-0.407, *df*=42, *P*=0.69**

Supplementary Table 2. Summary of model fits for the different evolutionary models tested in this study for each physiological, morphological, and life history trait in phrynosomatid lizards. O= oviparous and V= viviparous. BM is a single-rate Brownian motion model. OU1 is a single-peak, single-rate Ornstein-Uhlenbeck model. OUM is a two-peak, single-rate Ornstein-Uhlenbeck model. Models with better support are shown in bold. These analyses were conducted with 500 simulations across our maximum clade credibility tree. % represents the percentage of simulations supporting each evolutionary model. For the best-fitting model, we provide the rate of stochastic trait evolution (σ^2), and the resulting evolutionary optimal trait value(s) (θ) unless the best-fitting model was the BM model. Within parentheses we included confidence intervals (α 0.05).

**Trait**	**BM**			**OU1**			**OUM**			**Best-fitting model**	
	ΔAICc	Weight	%	ΔAICc	Weight	%	ΔAICc	Weight	%	σ2	θ
Critical thermal minimum *n*= 36 O and 27 V	21.19	<0.001	0	8.88	0.001	0	**0**	**0.99**	**100**	19.8	13.008 (13.006-13.011) O and 9.95 (9.94-9.96) V
Body temperature *n*= 63 O and 38 V	21.4	<0.001	0	6.8	0.03	0	**0**	**0.97**	**100**	0.5537	34.874 (34.871-34.876) O and 29.74 (29.73-29.76) V
Preferred body temperature *n*=48 O and 32 V	16.23	<0.001	0	2.72	0.2	1	**0**	**0.8**	**99**	0.3959	34.612 (34.611-34.613) O and 31.9 (31.88-31.91) V
Critical thermal maximum *n*= 40 O and 27 V	14.5	<0.001	0	3.81	0.13	9	**0**	**0.87**	**91**	0.5238	41.936 (41.93-41.94) O and 37.56 (37.5-37.62) V
Mass-specific metabolic rate *n*= 59 O and 36 V	8.15	0.01	0	2	0.27	41	**0**	**0.72**	**59**	1.49e-06	0.019808 (0.019805-0.019811) O and 0.01078 (0.01074-0.01083) V

Mass-corrected metabolic rate *n*= 39 O and 26 V	7.84	0.02	0	2.47	0.22	7	**0**	**0.76**	**93**	0.003	-3.4181 (-3.4186 - -3.4176) O and -3.837 (-3.840 – 3.833) V
Inactivity mass-corrected metabolic rate *n*= 20 O and 22 V	20.6	<0.001		3.78	0.02	6	**0**	**0.87**	**99.8**	4.58	-4.56 (-4.5593 - -4.5607) O and -5.0591 (-5.0587 - -5.0596) V
Temperature-corrected metabolic rate *n*= 24 O and 14 V	2.14	0.2	0	**0**	**0.58**	**99.8**	1.89	0.22	0.02	0.026	19.66 both
Adult body mass *n*= 74 O and 42 V	**0**	**0.43**	**100**	0.097	0.41	0	1.96	0.16	0	0.0045	-
Adult body size *n*= 74 O and 42 V	**1.44**	**0.26**	**100**	0	0.53	0	1.84	0.21	0	0.0006	-
Offspring mass *n*= 54 O and 23 V	2.19	0.2	0	**0**	**0.6**	**100**	2.15	0.2	0	0.0031	0.83 both
Offspring size *n*= 40 O and 25 V	4.81	0.06	0	**0**	**0.68**	**100**	1.88	0.26	0	0.0004	26.4 both
Annual fecundity *n*= 64 O and 36 V	15.25	<0.001	0	5.39	0.06	1	**0**	**0.94**	**99**	0.005	9.718 (9.715-9.721) O and 3.89 (3.87-3.91) V
Mass-specific production *n*= 54 O and 23 V	28.9	<0.001	0	10.04	0.006	**0**	**0**	**0.99**	**100**	0.223	1.0122 (1.0118-1.0125) O and 0.421 (0.419-0.422) V

Supplementary Table 3. Summary of the model fits for the different evolutionary models tested in this study for each physiological, morphological and life history trait in phrynosomatid lizards. O= oviparous and V= viviparous. BM is a single-rate Brownian motion model. OU1 is a single-peak, single-rate Ornstein-Uhlenbeck model. OUM is a two-peak, single-rate Ornstein-Uhlenbeck model. Models with better support are shown in bold. These analyses were conducted by performing one simulation across 500 individually-sampled trees from the posterior distribution. % represents the percentage of simulations supporting each evolutionary model. For the best-fitting model, we provide the rate of stochastic trait evolution (σ^2), and the resulting evolutionary optimal trait value(s) (θ) unless the best-fitting model was the BM model. Within parentheses we included confidence intervals (α 0.05).

**Trait**	**BM**			**OU1**			**OUM**			**Best-fitting model**	
	ΔAICc	Weight	%	ΔAICc	Weight	%	ΔAICc	Weight	%	σ2	θ
Critical thermal minimum *n*= 36 O and 27 V	24.3	<0.001	0	8.9	0.01	0	**0**	**0.99**	**100**	26.9	12.999 (12.998-13.001) O and 9.984 (9.979-9.988) V
Body temperature *n*= 63 O and 38 V	24.9	<0.001	0	7.2	0.02	0	**0**	**0.98**	**100**	1.98	34.838 (34.834-34.843) O and 29.85 (29.82-29.88) V
Preferred body temperature *n*=48 O and 32 V	19.2	<0.001	0	3.5	0.15	0.2	**0**	**0.85**	**99.8**	0.737	34.67 (34.667-34.673) O and 32.16 (32.14-32.17) V
Critical thermal maximum *n*= 40 O and 27 V	20.4	<0.001	0	4.9	0.08	1	**0**	**0.92**	**99**	2.2	41.84 (41.83-41.85) O and 37.74 (37.68-37.79) V
Mass-specific metabolic rate *n*= 59 O and 36 V	12.9	0.001	0	3.1	0.17	28	**0**	**0.82**	**72**	7.6e-06	0.019708 (0.019693-0.019723) O and 0.01074 (0.01069-0.01080) V

Mass-corrected metabolic rate<br>*n*= 39 O and 26 V	8.91	0.009	0	2.62	0.21	25	**0**	**0.78**	**75**	0.005	-3.421 (-3.4216 - -3.4206) O and -3.853 (-3.857 - -3.849) V
Inactivity mass-corrected metabolic rate<br>n= 20 O and 22 V	20.2	<0.001	0	3.2	0.17	6	**0**	**0.83**	**94**	4.62	-4.5665 (-4.5653 - -4.5676) O and -5.0567 (-5.0562 - -5.0572) V
Temperature-corrected metabolic rate<br>*n*= 24 O and 14 V	2.69	0.16	39	**0**	**0.60**	**61**	1.88	0.24	0	0.03	19.66 both
Adult body mass<br>*n*= 74 O and 42 V	3	0.14	74	**0**	**0.61**	**26**	1.7	0.26	0	0.016	8.43 both
Adult body size<br>*n*= 74 O and 42 V	4.7	0.06	36	**0**	**0.66**	**64**	1.7	0.28	0	0.001	61.1 both
Offspring mass<br>*n*= 54 O and 23 V	2.8	0.15	29	**0**	**0.64**	**71**	2.1	0.21	0	0.003	0.83 both
Offspring size<br>*n*= 40 O and 25 V	9	0.008	4	**0**	**0.72**	**96**	2	0.27	0	4.36	26.7 both
Annual fecundity<br>*n*= 64 O and 36 V	17.7	<0.001	0	3.9	0.12	0.2	**0**	**0.88**	**99.8**	0.008	9.76 (9.75-9.77) O and 4.06 (4.02-4.09) V
Mass-specific production<br>*n*= 54 O and 23 V	30.8	<0.001	0	10.5	0.005	0	**0**	**0.99**	**100**	0.66	1.019 (1.0186-1.0193) O and 0.45 (0.448-0.451) V

Supplementary Table 4. Results from phylogenetic general least squares (PGLS) analyses assessing the relationship between morphological and life history traits, and between thermal physiological traits and thermal environment in phrynosomatid lizards. O= oviparous and V= viviparous. CT_{min}= critical thermal minimum, T_b= field body temperature, T_{pref}= preferred body temperature, CT_{max}= critical thermal maximum, MAT= mean annual temperature, MTWQ= mean temperature of the warmest quarter, MTCQ= mean temperature of the coldest quarter, and T_e= operative temperatures. The sample size for each analysis is given in parentheses. When we tested for differences in slope or intercept among parity modes, we consider statistical significance with a *P*-value below 0.025 (Bonferroni-corrected significance level).

**Trait (*n*)**	**Slope±*SE***	**Intercept±*SE***	***df***	***P***
Body mass ~ Body size (18 O and 12 V)	0.2883±0.02	1.5218±0.03	28	**<0.001**
Body mass ~ Body size + parity mode (18 O and 12 V)	0.2886±0.02	1.522±0.03	27	0.86
Body mass ~ Body size * parity mode (18 O and 12 V)	0.2833±0.03	1.5265±0.03	26	0.7
Clutch/litter size ~ SVL females (64 O and 36 V)	1.1321±0.18	-1.2362±0.34	98	**<0.001**
Clutch/litter size ~ SVL females + parity mode (64 O and 36 V)	1.116±0.18	-1.1991±0.33	97	0.09
Clutch/litter size ~ SVL females * parity mode (64 O and 36 V)	1.046±0.24	-1.0742±0.45	96	0.7
SVL neonates ~ SVL females (39 O and 25 V)	0.2893±0.07	0.9064±0.13	62	**<0.001**
SVL neonates ~ SVL females + parity mode (39 O and 25 V)	0.291±0.07	0.9024±0.13	61	0.7
SVL neonates ~ SVL females * parity mode (39 O and 25 V)	0.311±0.1	0.8661±0.18	60	0.8
CT_{min} ~ MAT (36 O and 26 V)	0.354±0.07	5.639±2.6	60	**<0.001**
CT_{min} ~ MAT + parity mode (36 O and 26 V)	-1.395±1.4	5.943±2.6	59	0.3
CT_{min} ~ MAT * parity mode (36 O and 26 V)	-0.206±0.13	3.542±3	58	0.12
CT_{min} ~ MTWQ (36 O and 26 V)	0.31±0.07	5.164±3	60	**<0.001**
CT_{min} ~ MTWQ + parity mode (36 O and 26 V)	-0.6909±1.5	5.446±3.1	59	0.65
CT_{min} ~ MTWQ * parity mode (36 O and 26 V)	-0.1513±0.14	3.105±3.8	58	0.29
CT_{min} ~ MTCQ (36 O and 26 V)	0.3071±0.06	7.91±2.5	60	**<0.001**
CT_{min} ~ MTCQ + parity mode (36 O and 26 V)	-2.2117±1.4	8.1573±2.4	59	0.1
CT_{min} ~ MTCQ * parity mode (36 O and 26 V)	-0.1443±0.1	6.9789±2.6	58	0.2
CT_{min} ~ T_e (29 O and 25 V)	0.1698±0.06	7.2±2.93	52	**0.005**
CT_{min} ~ T_e + parity mode (29 O and 25 V)	-1.0619±1.54	7.67±3.02	51	0.49

CT_{min} ~ T_e * parity mode (29 O and 25 V)	0.0345±0.13	8.46±4.2	50	0.8
T_b ~ MAT (55 O and 37 V)	0.165±0.04	31.49±1.8	90	**<0.001**
T_b ~ MAT + parity mode (55 O and 37 V)	-2.118±0.87	31.88±1.7	89	**0.018**
T_b ~ MAT * parity mode (55 O and 37 V)	-0.164±0.07	30.52±1.8	88	**0.02**
T_b ~ MAT (55 O)	0.230±0.04	30.30±1.6	53	**<0.001**
T_b ~ MAT (37 V)	0.055±0.06	30.69±2.6	35	0.38
T_b ~ MTWQ (55 O and 37 V)	0.1902±0.04	30.08±1.8	90	**<0.001**
T_b ~ MTWQ + parity mode (55 O and 37 V)	-1.5375±0.9	30.62±1.8	89	0.08
T_b ~ MTWQ * parity mode (55 O and 37 V)	-0.1503±0.07	28.8±2	88	0.03
T_b ~ MTCQ (55 O and 37 V)	0.1129±0.03	33.11±1.7	90	**0.002**
T_b ~ MTCQ + parity mode (55 O and 37 V)	-2.5686±0.89	33.31±1.7	89	**0.005**
T_b ~ MTCQ * parity mode (55 O and 37 V)	-0.1721±0.07	32.39±1.7	88	**0.01**
T_b ~ MTCQ (55 O)	0.177±0.04	32.32±1.5	53	**<0.001**
T_b ~ MTCQ (37 V)	0.0047±0.06	31.5±2.5	35	0.9
T_b ~ T_e (35 O and 32 V)	0.1342±0.03	30.46±1.7	65	**<0.001**
T_b ~ T_e + parity mode (35 O and 32 V)	-2.42±0.88	31.34±1.7	64	**0.007**
T_b ~ T_e * parity mode (35 O and 32 V)	-0.1823±0.06	26.96±2.2	63	0.05
T_b ~ T_e (35 O)	0.2714±0.04	26.28±1.6	33	**<0.01**
T_b ~ T_e (32 V)	0.0667±0.04	29.44±2.5	30	0.1
T_{pref} ~ MAT (47 O and 32 V)	-0.0202±0.04	35.05±1.5	77	0.6
T_{pref} ~ MAT + parity mode (47 O and 32 V)	-1.0519±0.78	35.27±1.5	76	0.18
T_{pref} ~ MAT * parity mode (47 O and 32 V)	-0.1114±0.07	34±1.7	75	0.13
T_{pref} ~ MTWQ (47 O and 32 V)	-0.0124±0.04	34.94±1.6	77	0.7
T_{pref} ~ MTWQ + parity mode (47 O and 32 V)	-1.1248±0.8	35.4±1.6	76	0.16
T_{pref} ~ MTWQ * parity mode (47 O and 32 V)	-0.1139±0.07	33.64±2	75	0.12
T_{pref} ~ MTCQ (47 O and 32 V)	-0.0202±0.03	34.95±1.4	77	0.55
T_{pref} ~ MTCQ + parity mode (47 O and 32 V)	-0.9779±0.77	35.03±1.4	76	0.21
T_{pref} ~ MTCQ * parity mode (47 O and 32 V)	0.0826±0.06	34.41±1.5	75	0.21
T_{pref} ~ T_e (35 O and 28 V)	0.0333±0.03	33.35±1.49	61	0.2
T_{pref} ~ T_e + parity mode (35 O and 28 V)	-1.0719±0.8	33.75±1.52	60	0.18
T_{pref} ~ T_e * parity mode (35 O and 28 V)	-0.0666±0.06	32.2±2.06	59	0.27

CT_{max} ~ MAT (37 O and 26 V)	0.032±0.05	41.29±2	61	0.52
CT_{max} ~ MAT + parity mode (37 O and 26 V)	-1.295±1.09	41.55±1.98	60	0.24
CT_{max} ~ MAT * parity mode (37 O and 26 V)	-0.164±0.09	39.82±2.2	59	0.08
CT_{max} ~ MTWQ (37 O and 26 V)	0.0815±0.05	39.95±2.1	61	0.11
CT_{max} ~ MTWQ + parity mode (37 O and 26 V)	-1.0183±1.1	40.32±2.1	60	0.36
CT_{max} ~ MTWQ * parity mode (37 O and 26 V)	-0.2266±0.09	37.15±2.4	59	**0.02**
CT_{max} ~ MTWQ (37 O)	0.192±0.07	37.41±2.2	35	**0.007**
CT_{max} ~ MTWQ (26 V)	-0.0269±0.07	40.91±3	24	0.7
CT_{max} ~ MTCQ (37 O and 26 V)	-0.036±0.04	42.47±1.8	61	0.4
CT_{max} ~ MTCQ + parity mode (37 O and 26 V)	-1.333±1.08	42.61±1.8	60	0.2
CT_{max} ~ MTCQ * parity mode (37 O and 26 V)	-0.0595±0.09	42.17±1.9	59	0.5
CT_{max} ~ T_e (30 O and 25 V)	0.0332±0.04	40.68±1.9	53	0.39
CT_{max} ~ T_e + parity mode (30 O and 25 V)	-0.8284±1.02	41.05±2	52	0.42
CT_{max} ~ T_e * parity mode (30 O and 25 V)	-0.1632±0.08	37.3±2.68	51	0.05
CT_{max} ~ T_e (30 O)	0.1207±0.06	38.13±2.1	28	**0.04**
CT_{max} ~ T_e (25 V)	-0.0126±0.06	40.20±3	23	0.8

Supplementary Table 5. Comparison of evolutionary and optimal regressions of thermal physiological traits in response to thermal variables, life-history traits, and body size. Values for phylogenetic half-life ($t_{1/2}$) in millions of years (phrynosomatid tree length= 57.14 millions of years), and rate of adaptation (α) for each thermal physiological trait also are included in the table. CT_{min}= critical thermal minimum, T_b= field-body temperature, T_{pref}= preferred body temperature, CT_{max}= critical thermal maximum, MAT= mean annual temperature, MTWQ= mean temperature of the warmest quarter, and MTCQ= mean temperature of the coldest quarter. O= oviparous, and V= viviparous. The sample size for each analysis is given in parentheses.

					**Evolutionary**		**Optimal**			
**Response traits**	**Predictor traits**	**Parity mode (*n*)**	$t_{1/2}$	α	**Intercept ± *SE***	**Slope ± *SE***	**Intercept ± *SE***	**Slope ± *SE***	R^2	**AICc**
CT_{min}	MAT	O (36)	0	∞	6.12 ± 1.6	0.359 ± 0.08	6.12 ± 1.6	0.359 ± 0.08	0.35	184
		V (26)	0	∞	4.06 ± 1.7	0.345 ± 0.09	4.06 ± 1.7	0.345 ± 0.09	0.35	130.5
CT_{min}	MTWQ	O (36)	0	∞	6.9 ± 2.6	0.25 ± 0.1	6.9 ± 2.6	0.25 ± 0.1	0.14	194.6
		V (26)	0	∞	3.98 ± 2	0.307 ± 0.1	3.98 ± 2	0.307 ± 0.1	0.27	133.5
CT_{min}	MTCQ	O (36)	0	∞	8.78 ± 0.9	0.3 ± 0.06	8.78 ± 0.9	0.3 ± 0.06	0.41	180.5
		V (26)	0	∞	5.53 ± 1.4	0.31 ± 0.09	5.53 ± 1.4	0.31 ± 0.09	0.32	132
T_b	MAT	O (55)	13.8	0.05	30.55 ± 1.06	0.219 ± 0.05	30.55 ± 1.06	0.326 ± 0.07	0.28	238.1
		V (37)	8.5	0.08	30.39 ± 1.1	0.0608 ± 0.06	30.39 ± 1.1	0.0772 ± 0.08	0.02	156.7
T_b	MTWQ	O (55)	10.6	0.07	28.07 ± 1.4	0.2682 ± 0.05	28.07 ± 1.4	0.3627 ± 0.07	0.33	233.6
		V (37)	6.4	0.11	28.83 ± 1.2	0.1307 ± 0.06	28.83 ± 1.2	0.1559 ± 0.07	0.13	152.7
T_b	MTCQ	O (55)	16.5	0.04	32.73 ± 0.8	0.1491 ± 0.04	32.73 ± 0.8	0.2401 ± 0.06	0.2	245
		V (37)	7.6	0.09	31.53 ± 0.9	-0.0116 ± 0.06	31.53 ± 0.9	-0.0144 ± 0.07	0.001	157.6
T_{pref}	MAT	O (47)	9.7	0.07	33.66 ± 1.1	0.051 ± 0.05	33.66 ± 1.1	0.0673 ± 0.07	0.02	186.7
		V (32)	13.8	0.05	34.57 ± 1.2	-0.0946 ± 0.06	34.57 ± 1.2	-0.1411 ± 0.09	0.07	133.3
T_{pref}	MTWQ	O (47)	0.8	0.87	31.62 ± 1.3	0.1245 ± 0.05	31.62 ± 1.3	0.1269 ± 0.05	0.11	182
		V (32)	15.3	0.05	34.74 ± 1.31	-0.0869 ± 0.06	34.74 ± 1.31	-0.1352 ± 0.09	0.07	133.6
T_{pref}	MTCQ	O (47)	11.1	0.06	34.6 ± 0.66	0.005 ± 0.04	34.6 ± 0.66	0.0069 ± 0.05	0.0003	187.7
		V (32)	13.7	0.05	34.02 ± 0.96	-0.0775 ± 0.05	34.02 ± 0.96	-0.1151 ± 0.08	0.06	133.8
CT_{max}	MAT	O (37)	17.8	0.04	40.67 ± 1.5	0.0581 ± 0.07	40.67 ± 1.5	0.0969 ± 0.1	0.02	176.1
		V (26)	8.8	0.08	40.07 ± 1.4	-0.033 ± 0.08	40.07 ± 1.4	-0.0425 ± 0.1	0.007	119.9
CT_{max}	MTWQ	O (37)	20	0.03	38.43 ± 1.9	0.1414 ± 0.07	38.43 ± 1.9	0.2506 ± 0.13	0.09	173
		V (26)	8.9	0.08	39.6 ± 1.6	-0.0044 ± 0.07	39.6 ± 1.6	-0.0057 ± 0.09	0.0001	120
CT_{max}	MTCQ	O (37)	15.5	0.04	41.95 ± 1	-0.0121 ± 0.06	41.95 ± 1	-0.0189 ± 0.09	0.001	176.8
		V (26)	8.7	0.08	40.25 ± 1.1	-0.0538 ± 0.07	40.25 ± 1.1	-0.0688 ± 0.09	0.02	119.4

Supplementary Table 6. Summary of Wheatsheaf indices (*w*) tested in this study for each physiological, morphological and life history trait in phrynosomatid lizards. Within parentheses we included confidence intervals (two-sided α 0.05).

Trait	*w*	*P*
Critical thermal minimum (*n*= 36 O and 27 V)	1.02 (1 – 1.08)	**0.006**
Body temperature (*n*= 63 O and 38 V)	1.1 (1.08 – 1.12)	**<0.001**
Preferred body temperature (n=48 O and 32 V)	0.84 (0.83 – 0.86)	0.2
Critical thermal maximum (*n*= 40 O and 27 V)	0.96 (0.94 – 1.02)	**0.01**
Mass-specific metabolic rate (*n*= 59 O and 36 V)	1.38 (1.36 – 1.42)	**<0.001**
Adult body mass (*n*= 74 O and 42 V)	0.85 (0.83 – 0.88)	0.05
Adult body size (*n*= 74 O and 42 V)	0.84 (0.82 – 0.87)	0.06
Offspring mass (*n*= 54 O and 23 V)	0.92 (0.88 – 0.96)	0.18
Offspring size (*n*= 40 O and 25 V)	0.97 (0.93 – 1.04)	0.1
Annual fecundity (*n*= 64 O and 36 V)	0.97 (0.95 – 1)	**0.01**
Mass-specific production (*n*= 54 O and 23 V)	2.52 (2.4 – 2.65)	**<0.001**

Supplementary Table 7. Summary of the model fits for the different evolutionary models tested in this study for phrynosomatid lizards. Here, some analyses included in Supplementary table 2 were re-conducted to include data of adult body size (44.08 mm), adult body mass (2.88 g) and annual fecundity (8.25 neonates) of *Sceloporus goldmani* (considering it as a viviparous species)[1,2]; see Methods; Data collection; Parity mode, for more details. O= oviparous and V= viviparous. BM is a single-peak, single-rate Brownian motion model. OU1 is a single-peak, single-rate Ornstein-Uhlenbeck model. OUM is a two-peak, single-rate Ornstein-Uhlenbeck model. Models with better support are shown in bold. These analyses were conducted with 500 simulations across our maximum clade credibility tree. For the best-fitting model, we provide the rate of stochastic trait evolution (σ^2), and the resulting evolutionary optimal trait value(s) (θ) unless the best-fitting model was the BM model.

**Trait**	**BM**		**OU1**		**OUM**		**Best-fitting model**	
	ΔAICc	Weight	ΔAICc	Weight	ΔAICc	Weight	σ2	θ
Adult body size *n*= 74 O and 43 V	1.26	0.28	**0**	**0.52**	1.85	0.2	0.0006	61.1 both
Adult body mass *n*= 74 O and 43 V	**0**	**0.45**	0.24	0.4	2.1	0.15	0.0045	-
Annual fecundity *n*= 65 O and 36 V	15.15	<0.001	5.32	0.07	**0**	**0.93**	0.005	9.7 O and 3.9 V

References

1. Smith, H. M. & Hall, W. P. Contributions to the concepts of reproductive cycles and the systematics of the Scalaris group of the lizards genus *Sceloporus*. *Gt. Basin Nat.* **34**, 97–104 (1974).

2. Carbajal-Márquez, R. A. & Quintero-Díaz, G. E. Natural history of *Sceloporus goldmani* (Squamata: Phrynosomatidae) in its southern distribution. *Herpetol. Notes* **10**, 161–167 (2017).
