# Supplementary material for: Exceptional parallelisms characterize the evolutionary transition to live birth in phrynosomatid lizards
Source: Nat Commun. 2022 May 24;13:2881. doi: 10.1038/s41467-022-30535-w (PMC9130271; doi:10.1038/s41467-022-30535-w)
Supplement: Supplementary file 3 — Description of Additional Supplementary Files [file 41467_2022_30535_MOESM3_ESM.pdf]

Description of Additional Supplementary Files

File Name: Supplementary Data 1
Description: Parity mode, thermoregulatory behavior, and thermal physiology, modelled metabolic rate, morphology and life history traits, and massspecific production for phrynosomatid lizards.

File Name: Supplementary Data 2
Description: GenBank accession numbers associated with five mitochondrial and eight nuclear genes for phrynosomatid species.

File Name: Supplementary Data 3
Description: Concatenated genetic matrix of 9,837 bp for 149 phrynosomatid species.

File Name: Supplementary Data 4
Description: Ultrametric tree of phrynosomatids.

File Name: Supplementary Data 5
Description: 500 trees, randomly sampled from our posterior distribution.

File Name: Supplementary Data 6
Description: Code used to perform the evolutionary analyses.
