# Supplementary material for: Exceptional parallelisms characterize the evolutionary transition to live birth in phrynosomatid lizards
Source: Nat Commun. 2022 May 24;13:2881. doi: 10.1038/s41467-022-30535-w (PMC9130271; doi:10.1038/s41467-022-30535-w)
Supplement: Supplementary file 7 — Reporting Summary [file 41467_2022_30535_MOESM7_ESM.pdf]

nature research

Corresponding author(s): Saúl F. Domínguez-Guerrero

Last updated by author(s): Apr 30, 2022

# Reporting Summary

Nature Research wishes to improve the reproducibility of the work that we publish. This form provides structure for consistency and transparency in reporting. For further information on Nature Research policies, see our Editorial Policies and the Editorial Policy Checklist.

## Statistics

For all statistical analyses, confirm that the following items are present in the figure legend, table legend, main text, or Methods section.

| n/a | Confirmed | |
|---|---|---|
| ☐ | ☒ | The exact sample size (*n*) for each experimental group/condition, given as a discrete number and unit of measurement |
| ☐ | ☒ | A statement on whether measurements were taken from distinct samples or whether the same sample was measured repeatedly |
| ☐ | ☒ | The statistical test(s) used AND whether they are one- or two-sided<br>*Only common tests should be described solely by name; describe more complex techniques in the Methods section.* |
| ☐ | ☒ | A description of all covariates tested |
| ☐ | ☒ | A description of any assumptions or corrections, such as tests of normality and adjustment for multiple comparisons |
| ☐ | ☒ | A full description of the statistical parameters including central tendency (e.g. means) or other basic estimates (e.g. regression coefficient) AND variation (e.g. standard deviation) or associated estimates of uncertainty (e.g. confidence intervals) |
| ☐ | ☒ | For null hypothesis testing, the test statistic (e.g. *F*, *t*, *r*) with confidence intervals, effect sizes, degrees of freedom and *P* value noted<br>*Give P values as exact values whenever suitable.* |
| ☐ | ☒ | For Bayesian analysis, information on the choice of priors and Markov chain Monte Carlo settings |
| ☒ | ☐ | For hierarchical and complex designs, identification of the appropriate level for tests and full reporting of outcomes |
| ☒ | ☐ | Estimates of effect sizes (e.g. Cohen's *d*, Pearson's *r*), indicating how they were calculated |

*Our web collection on statistics for biologists contains articles on many of the points above.*

## Software and code

Policy information about availability of computer code

| | |
|---|---|
| Data collection | We did not use software to collect data. |
| Data analysis | To align and concatenate genes, as well as estimate the ultrametric tree, we used the softwares MAFFT (ver. 7), Mesquite (ver. 3.6), BEAST (ver. 2.5.2), Tracer (ver. 1.7), LogCombiner (ver. 2.5.2), TreeAnnotator (ver. 1.8.4), and the portal CIPRES. To georeferenced coordinates of populations for some phrynosomatids we used Google Earth Pro (ver. 7.3.3). We conducted all evolutionary analyses using R environment for statistical computing (ver. 3.6.0 and 4.1.1), and the R packages phytools (ver. 0.6.99), OUwie (ver. 1.57), windex (ver. 2.0.2), nlme (ver. 3.1.139), phylolm (ver. 2.6.2), and slouch (ver. 2.1.2). We generated the graphics using the R package ggplot2 (ver. 3.2.1), and we edited the figures using Adobe Illustrator. |

For manuscripts utilizing custom algorithms or software that are central to the research but not yet described in published literature, software must be made available to editors and reviewers. We strongly encourage code deposition in a community repository (e.g. GitHub). See the Nature Research guidelines for submitting code & software for further information.

## Data

Policy information about availability of data

All manuscripts must include a data availability statement. This statement should provide the following information, where applicable:

- Accession codes, unique identifiers, or web links for publicly available datasets
- A list of figures that have associated raw data
- A description of any restrictions on data availability

The data (behavioral, life history traits, morphological and physiological) and the GenBank accession numbers compiled or generated in this study, as well the code used for the evolutionary analyses are provided in the Supplementary Data files.

# Field-specific reporting

Please select the one below that is the best fit for your research. If you are not sure, read the appropriate sections before making your selection.

☐ Life sciences ☐ Behavioural & social sciences ☒ Ecological, evolutionary & environmental sciences

For a reference copy of the document with all sections, see nature.com/documents/nr-reporting-summary-flat.pdf

# Ecological, evolutionary & environmental sciences study design

All studies must disclose on these points even when the disclosure is negative.

| | |
|---|---|
| Study description | We used previously published and newly collected data of reproductive parity mode, thermoregulatory behavior, thermal physiology, modelled metabolic rate, morphology, life history traits, and mass-specific production for 125 phrynosomatid species, to test apart evolutionary convergences during the transitions from egg-laying to live birth. |
| Research sample | Our dataset encompasses information for 125 phrynosomatid species (80 oviparous and 45 viviparous species), which represents 73% of all phrynosomatids. For that data base, we obtained newly collected data for 68 species (41 oviparous and 27 viviparous species). For previously published data, we limited our selection to species that were measured with similar methods. For newly collected data of thermoregulatory behavior and thermal physiology we sampled adult females and males (we did not include gravid females in experiments of thermal tolerances). For newly collected data of adult body size and adult body mass we included only females. |
| Sampling strategy | All phrynosomatid species for which we were able to obtain newly collected or previously published data were included in this study. For the newly collected data, we performed field work through latitudinal (31.921 °N to 16.032 °N) and altitudinal gradients (2-4151 m), to obtain data of thermoregulatory behavior and thermal physiology through natural thermal gradients. Thus, we collected data in places with mean annual temperature from 7°C to 28°C. To obtain data in each population we did not perform sample-size calculation; instead, we collected data for the maximum of individuals that we can catch (always in accordance with collecting permits) during our field work. |
| Data collection | S.F.D.-G. recorded all data (newly collected or previously published) in an Excel spreadsheet, which we supply as Supplementary Data 1 |
| Timing and spatial scale | We performed field trips from 2008 to 2019 to obtain new data, and we performed Google Scholar searches (weekly) from March 2019 to March 2020 to obtain published information. We assembled our dataset between January 2020 and March 2020, gathering the newly collected and the previously published data. |
| Data exclusions | No data were excluded for the analyses. |
| Reproducibility | All evolutionary analyses were performed in triplicate, with qualitatively similar results. |
| Randomization | For newly collected data of body temperature, adult body mass and adult body size, we measured the data when we collected the lizards. Therefore a randomization procedure was not relevant. To obtain newly collected data of preferred body temperature, we placed randomly each lizard into every track of the thermal gradient, and to obtain newly collected data of thermal tolerances, we placed randomly each individual into the plastic containers where we performed the experiments. |
| Blinding | To obtain newly collected data of field body temperature, preferred body temperature, and thermal tolerances, we used standardized (published) protocols, and digital thermometers; therefore blinding was not relevant. For example, we measured field body temperature (cloacal temperature) of lizards as quick as possible (<10s) after we captured them, or we measured critical thermal minimum and maximum, as the minimum or maximal body temperature, respectively, at which individuals lose locomotion response. |

Did the study involve field work? ☒ Yes ☐ No

## Field work, collection and transport

| | |
|---|---|
| Field conditions | We performed our field work when phrynosomatids are active at their natural habitat. This was necessary to obtain the field body temperatures of activity of individuals. As phrynosomatids are diurnal and heliotermic lizards, we were catching individuals only during the day (not at night) and on non-rainy days. |
| Location | Data come from lizard populations in Mexico, from latitude 31.921 °N (Baja California) to 16.032 °N (Oaxaca), and from 2 to 4151 m elev. |
| Access & import/export | Permits supplied by the Dirección General de Vida Silvestre, México allowed us to conduct the field work presented in the study. The collecting permits were approved from 2008 to 2019, and are the following: SGPA/DGVS/07946/08, 03369/12, 00228/13, 07587/13, 01629/16, 01205/17, 02490/17, 06768/17, 000998/18, 002463/18, 002490/18, 002491/18, 003209/18, and 02523/19. |
| Disturbance | Our experiments were non-invasive and not harmful for lizards, which were subsequently released to their site of capture. |

# Reporting for specific materials, systems and methods

We require information from authors about some types of materials, experimental systems and methods used in many studies. Here, indicate whether each material, system or method listed is relevant to your study. If you are not sure if a list item applies to your research, read the appropriate section before selecting a response.

## Materials & experimental systems

| n/a | Involved in the study |
|---|---|
| ☒ | ☐ Antibodies |
| ☒ | ☐ Eukaryotic cell lines |
| ☒ | ☐ Palaeontology and archaeology |
| ☐ | ☒ Animals and other organisms |
| ☒ | ☐ Human research participants |
| ☒ | ☐ Clinical data |
| ☒ | ☐ Dual use research of concern |

## Methods

| n/a | Involved in the study |
|---|---|
| ☒ | ☐ ChIP-seq |
| ☒ | ☐ Flow cytometry |
| ☒ | ☐ MRI-based neuroimaging |

## Animals and other organisms

Policy information about studies involving animals; ARRIVE guidelines recommended for reporting animal research

| | |
|---|---|
| Laboratory animals | The study did not involve laboratory animals. |
| Wild animals | In this study we obtained newly collected data for 68 species of the genus Callisaurus, Cophosaurus, Holbrookia, Petrosaurus, Phrynosoma, Sceloporus, Uma, Urosaurus, and Uta. We captured only adult lizards (females and males) directly (with the hand) or by lasso. For laboratory experiments, animals were transported into individual bags to field laboratories. After each study in each population, animals were hydrated ad libitum and released to their habitat. |
| Field-collected samples | When we captured lizards, we maintained them in individual bags (to avoid stress), and the bags into a room at ~20 °C. At the next day we placed individuals into a thermal gradient during their activity time (typically from 8:00 to 17:00 h) to obtain preferred body temperatures. To obtain critical thermal limits, we maintained individuals at low and high temperatures for only for few minutes. When we maintained animals in captivity, we maintained their photoperiod similar to their natural habitat, and hydrated them after each experiment. Lizards were kept in laboratory no more than three days before being released back to the wild. |
| Ethics oversight | The data collection and experiments were conducted in accordance with the collecting permits (SGPA/DGVS/07946/08, 03369/12, 00228/13, 07587/13, 01629/16, 01205/17, 02490/17, 06768/17, 000998/18, 002463/18, 002490/18, 002491/18, 003209/18, and 02523/19) approved by Dirección General de Vida Silvestre, México. |

Note that full information on the approval of the study protocol must also be provided in the manuscript.
